# Global gene regulatory network underlying *miR165a* in Arabidopsis shoot apical meristem

**DOI:** 10.1038/s41598-023-49093-2

**Published:** 2023-12-14

**Authors:** Sonali Sinha, Sudeep Sahadevan, Carolyn Ohno, Hasthi Ram, Marcus G. Heisler

**Affiliations:** 1 National Institute of Plant Genome Research (NIPGR), Aruna Asaf Ali Marg, JNU Campus, New Delhi, 110067 India; 2 European Molecular Biology Laboratory, Meyerhofstrasse 1, Heidelberg, Germany; 3 School of Life and Environmental Sciences, University of Sydney, Sydney, NSW Australia

**Keywords:** Functional genomics, Gene expression profiling

## Abstract

Arabidopsis *microRNA165a (miR165a)* targets *Class III Homeodomain Leucine-Zipper (HD-ZIPIII)* transcription factors to regulate various aspects of plant development and stress response. Over-expression of *miR165a* mimics the loss-of-function phenotype of *HD-ZIPIII* genes and leading to ectopic organ formation, shoot apical meristem (SAM) termination, loss of leaf polarity, and defective vasculature development. However, the molecular mechanisms underlying these phenotypes remain unresolved. Here, we over-expressed *miR165a* in a dexamethasone inducible manner and identified differentially expressed genes in the SAM through RNA-Seq. Simultaneously, using multi-channel FACS combined with RNA-Seq approach, we characterized global transcriptome patterns in *miR165a* expressing cell-types compared to *HD-ZIPIII* expressing cell-types and other cell-types in SAM. By integrating our results we identified sets of genes which are up-regulated by miR165a as well have enriched expression in miR165a cell-types, and vice-versa. Known plant development related genes such as *HD-ZIPIII* and their targets *LITTLE ZIPPERs, Like AUXIN RESISTANT 2, BEL1-like homeodomain 6, ROTUNDIFOLIA like 16* were found to be down-regulated. Among the up-regulated genes, *GIBBERELLIN 2-OXIDASEs*, various elemental transporters (*YSL3, ZIFL1, SULTR*), and other transporter genes were prominent. Thus, the genes identified in this study help to unravel the molecular mechanism of *miR165a* and *HD-ZIPIII* regulated plant development and stress response.

## Introduction

In higher plants, such as Arabidopsis, adaxial-abaxial and radial (central-peripheral) polarity commence during the embryogenesis and it continue throughout the plant's lifespan. Many decades of past research has identified a plethora of genetic factors contributing to the organ polarity in embryo, Shoot Apical Meristem (SAM) and leaves. Among these genetic factors, the roles of *microRNA (miR) 165* and *miR166*, and *Class III* Homeodomain Leucine Zipper (HD-ZIPIII) have been very well established. These miRNAs express in the peripheral part of the SAM and at the abaxial side of leaves and target the transcripts of *HD-ZIPIII* genes, thereby restricting their expression in the central part of the SAM and at the adaxial side of leaves^1–5^. Knock-down of *miR165/166* through STTM (small tandem target mimic) approach causes up-regulation of *HD-ZIPIII* genes and pleotropic effects on plant development^6^. Conversely, over-expression of *miR165/166* results in reduced transcripts of *HD-ZIPIII* genes and phenotypes similar to loss-of-function mutant phenotypes of *HD-ZIPIII* genes, such as loss of polarity, loss of SAM and defective vascular tissues^7–9^. In addition to plant development, an additional role of *miR165/166* and *HD-ZIPIII* genes has also been discovered in drought stress and cold stress^10,11^. The PHB directly binds to the promoters of the *ABI4* and *BG1* promoters, which in turn regulates the ABA levels^10^. Recently, the role of *miR165/166-PHB* has also been shown in the heat stress and thermotolerance, where PHB directly represses the expression of heat stress transcription factors (*HSFA1s*), which globally regulate heat stress regulated genes^11^. This indicates that *miR165/166* and *HD-ZIPIII* genes together work in biological processes other than plant development. Thus, given the diverse role of *miR165/166* and their target genes, it is crucial to understand their downstream molecular mechanisms.

Earlier, transcriptomics studies were used to understand the global gene regulatory network downstream of an important *HD-ZIPIII* gene, *REVOLUTA (REV)*, after its ectopic over-expression in leaves and SAM^12,13^. However, these studies mainly explored the regulatory mechanism downstream of only *REV*, and fell short of identifying the complete gene regulatory network underlying *miR165/166* and complete set of *HD-ZIPIII* genes. A previous study performed global gene expression analysis through microarray after constitutive over-expression of *miR165a* on one-week-old seedling tissues and found genes involved in auxin signalling and vasculature development were affected^9^. However due to the nature of the experiments where *miR165a* was constitutively over-expressed, resulting in very strong developmental defects, it is likely that the identified genes were more reflective of phenotype differences between wild-type and transgenic lines, rather than immediate target genes.

To explore the function of *HD-ZIPIII* genes in SAM, earlier we had previously performed cell-type profiling to identify *REV* and *KANADI (KAN)* cell-type expressed genes, as well as identified *miR165a* and *REV* regulated genes after over-expressing *miR165a* (*pAtUBQ10*>>*miR165a*) and *REV (pAtML1*>>*REVr)*^12^. However, number of Differentially Expressed genes (DEGs) identified after over-expression of *miR165a* was much lower, compared with DEGs identified after epidermal REV expression. Secondly, the previous study didn’t identify *miR165a* cell-type expressed genes. To overcome these drawbacks, here in this study, using multi-colour FACS approach, we compared the gene expression profile in *miR165a* cells with *REV* expressing cells and other cell-types. Furthermore, we again identified *miR165a* regulated genes after over-expressing miR165a in SAM tissue. Integration of both sets of the data helped to identify the genes that are up/down-regulated after *miR165a* induction as well as have enriched/depleted expression in *miR165a* cells.

## Results

### Identification of *miR165a* regulated genes in Arabidopsis SAM

**Figure 1:**
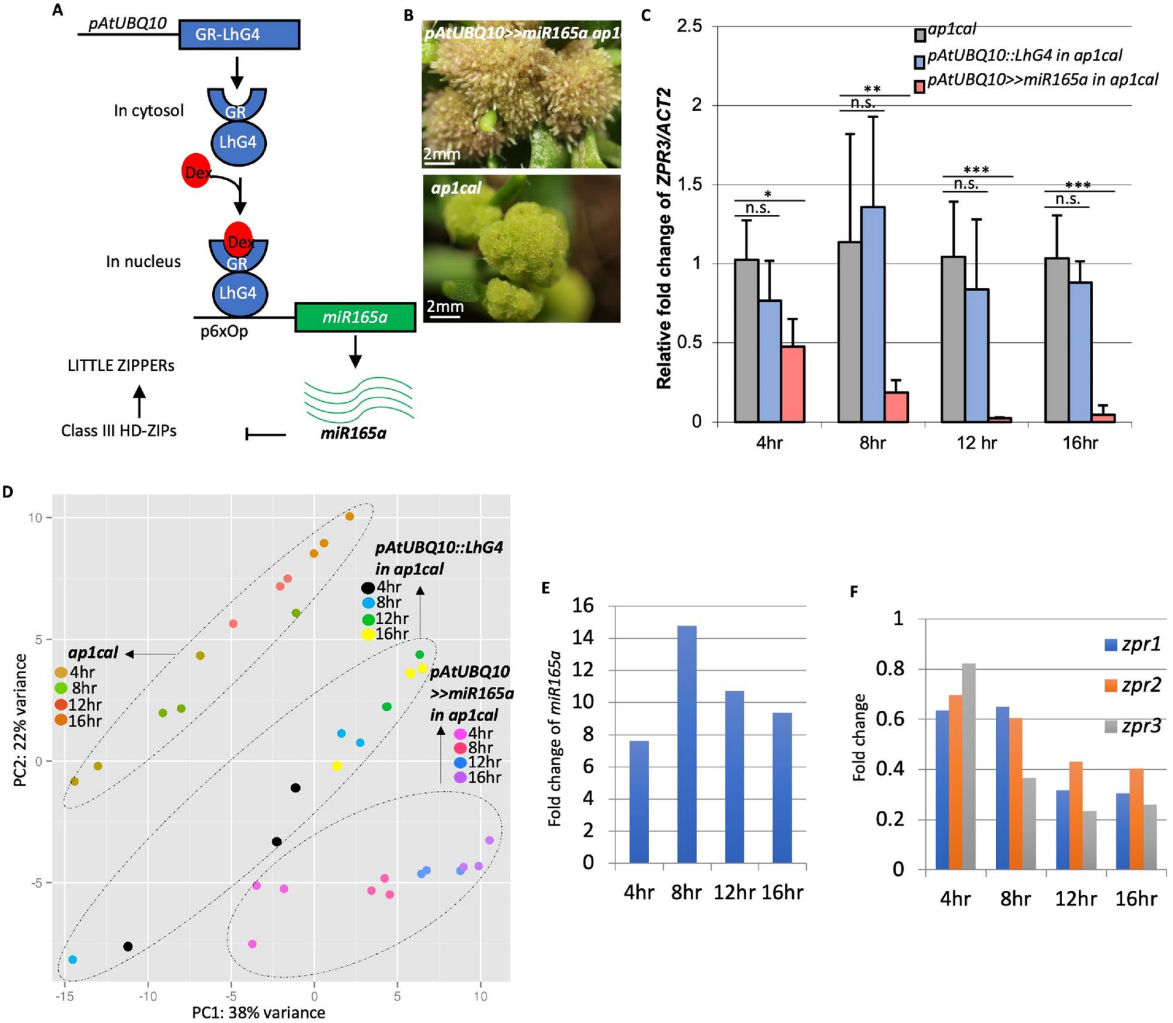
GR-LhG4 based RNA-Seq experiments successfully capture the global gene expression changes after *miR165a* induction. (**A**) Schematic of GR-LhG4 system used for Dex mediated induction of *miR165a*. (**B**) Phenotype of *ap1cal* meristems with (top picture) or without (bottom picture) induction of *pAtUBQ10*>>*miR165a* for 7 days. (**C**) Q-PCR analysis of *ZPR3* transcript after induction of *pAtUBQ10*>>*miR165a* or *pAtUBQ10::GR-LhG4* transgenes in *ap1cal* meristems. Asterisk indicates p-values, where *p-value < 0.05, **p-value < 0.01, ***p-value < 0.001. (**D**) Principal Component Analysis of RNA-Seq data. (**E**,**F**) Transcript levels of *miR165a* (**E**) and *ZPR1, ZPR2, ZPR3* (**F**) in *pAtUBQ10*>>*miR165a* line in comparison to *pAtUBQ10::GR-LhG4* in RNA-Seq data.

Expression of the *HD-ZIPIII* genes is repressed by *miR165/166* on the abaxial side of the leaf as well as on the periphery of the SAM. To further investigate the role of miRNA in SAM patterning, we aimed to identify miRNA165a regulated genes through RNA-Seq approach. For this purpose, we utilized a two-component dexamethasone inducible system to overexpress *miR165a*. Two expression cassettes, namely (1) *GR-LhG4* fusion protein^14^ driven by the Arabidopsis *UBIQUITIN 10 (AtUBQ10)* promoter (*pAtUBQ10*::*GR-LhG4*), and (2) the corresponding operator sequence (6x) of the LhG4 transcription factor placed upstream of *miR165a (p6xOp::miR165a)*, were placed on the same T-DNA (*pAtUBQ10*>>*miR165a*) (Fig. 1A). For control experiments, the *GR-LhG4* expression cassette alone (*pAtUBQ10*::*GR-LhG4* ) was also cloned into the T-DNA vector without the miR165a expression cassette. Both these DNA constructs were transformed separately into Arabidopsis *apetala1****-****1 cauliflower1****-****1* (*ap1cal)* mutants in order to enable bulk harvesting of meristematic tissue^15^. This system resulted in very high expression of *miR165a* upon dexamethasone (Dex) induction (Fig. 1E) and the formation of terminal outgrowths from the meristems (Fig. 1B). For RNA-Seq experiments, Dex treatment was done on SAMs of *pAtUBQ10*>>*miR165a ap1cal, pAtUBQ10*::*GR-LhG4 ap1cal* and *ap1cal* lines for 4 h, 8 h, 12 h and 16 h time-points, after which, SAM tissues were harvested.

Q-PCR analysis of *ZPR3* (target gene of class III HD-ZIPs) transcript revealed reduced levels in the *pAtUBQ10*>>*miR165a* line after induction indicating successful knock-down of class III HD-ZIP function (Fig. 1C). Principal Component (PC) analysis of RNA-Seq data revealed close clustering of biological replicates and different time-points separately for of each of the three lines (Fig. 1D). For the identification of Differentially Expressed Genes (DEGs), a criterion for log2 fold change (FC) > 1 and p-value < 0.05 was applied (Table 1).

**Table 1:** Number of differentially expressed genes in different comparisons after over-expression of *miR165a* and *LhG4*.

	*pAtUBQ10*>>*mir165a* in *ap1cal v*s. *pAtUB Q10::LhG4* in* ap1cal*		*pAtUBQ10*>>*mir165a* in *ap1cal v*s.* ap1cal*		*pAtUBQ10::LhG4* in *ap1cal v*s.* ap1cal*	
	Up	Down	Up	Down	Up	Down
4 h	9	29	68	21	55	1
8 h	59	31	100	35	41	1
12 h	54	68	129	40	84	6
16 h	46	68	171	63	63	0

Log2FC > 1 and adjusted p-value < 0.05 were used as a criteria for DEG consideration.

It was observed that LhG4 also regulates a relatively small number of genes most of which are up-regulated, consistent with the role of LhG4 as a transcriptional activator. Therefore to identify miR165a regulated genes, further analysis was focused on the differentially expressed genes (DEGs) specific to the *pAtUBQ10*>>*miR165a* line in comparison to *pAtUBQ10*::*GR-LhG4.* Surprisingly, among the five class *III HD-ZIP* genes, transcript levels of only *ATHB8* (only at 4 h) and *ATHB15* (at all time points) were found to be significantly reduced in the *pAtUBQ10*>>*miR165a* line, whereas no changes in transcript levels of *REV*, *PHB* and *PHV* were observed (Supp Table 1). Using Q-PCR approach, an earlier study has shown that all the five class III-HDZIPs are targeted by miR165a^9^, thus it seems that due to technical artifact *REV, PHB* and *PHV* were not identified as DEG. As miRNAs repress their target genes through either cleavage of target transcript or inhibition of translation of target transcript^16,17^, thus it may be possible that in addition to transcript cleavage the *REV*, *PHB* and *PHV* are also inhibited translationally. Consistent with this, *LITTLE ZIPPER (ZPR)1, ZPR2* and *ZPR3*, which are positively regulated by REV, PHB and PHV^18^, were down-regulated after *miR165a* induction (Fig. 1F).

### Functional annotations of *miR165a* regulated genes

**Figure 2:**
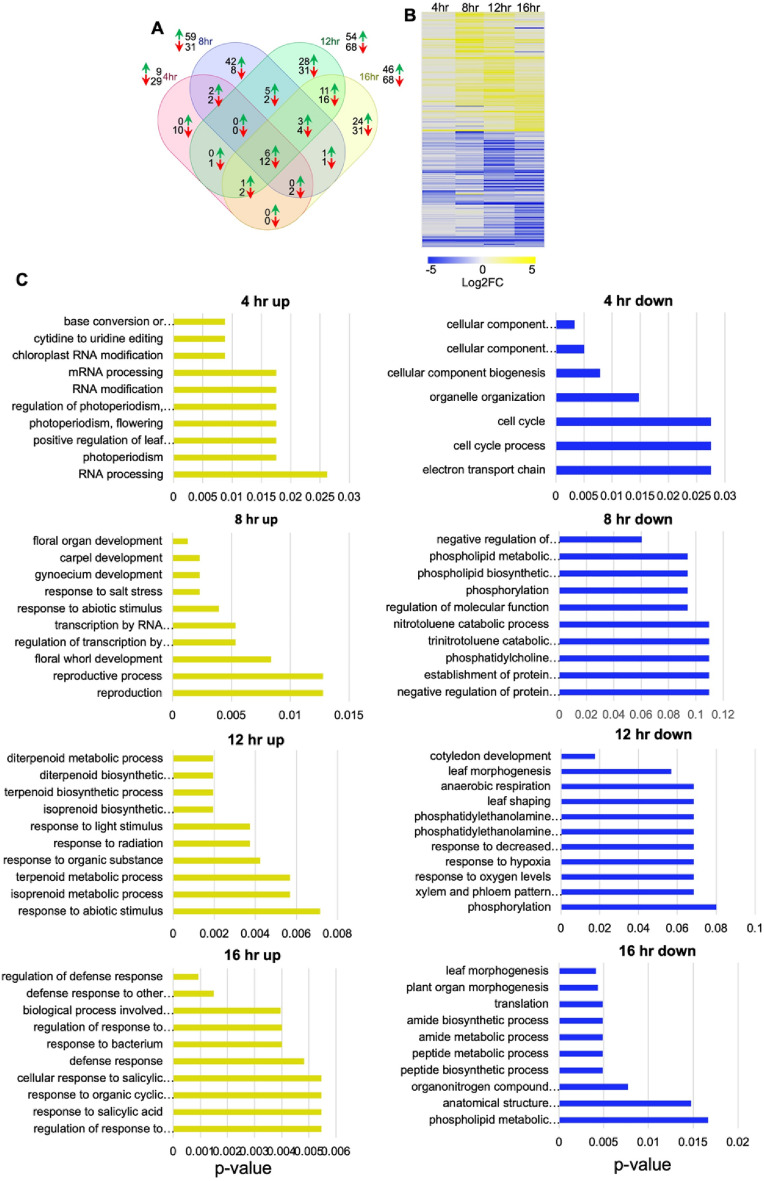
Analysis of transcriptome after induction of *pAtUBQ10*>>*miR165a* in *ap1cal* SAM. (**A**) Overlap analysis of differentially expressed genes in *pAtUBQ10*>>*miR165a* line against *pAtUBQ10::GR-LHG4* line at different time points. Green arrows represent up-regulated genes, and red arrows represent down-regulated genes. Numbers outside the Venn diagram show total number of up/down-regulated genes (**B**) Heatmap representation of log2 fold changes for all the differentially expressed genes at different time points. C. GO term enrichment of genes up/down-regulated at different time-points after induction of *pAtUBQ10*>>*miR165a*. Ten most significant GO terms are plotted here along with their p-values.

By analysing the overlap of DEGs at different time points, we identified sets of genes differentially expressed at individual time-points as well as genes differentially expressed across multiple time-points (Fig. 2A). Among all DEGs, six genes were up-regulated and 12 genes were down-regulated at all the time-points (Fig. 2A, Table 2).

**Table 2:** Genes up/down-regulated at all the time-points after *miRNA165a* induction, and genes with known role in plant development.

Gene ID	Description	Reported function	*pAtUBQ10*>>*miR165a*				miRNA cells Vs REV cells	
			4 h FC	8 h FC	12 h FC	16 h FC	Epidermis FC	Sub-epidermis FC
*AT1G01183*	miRNA165a	Represses Class III HD-ZIP gene expression	3.28	4.27	3.83	3.59		
*AT1G45120*	Transposable element gene		1.55	1.62	1.60	1.51		
*AT1G53260*	Hypothetical protein		1.47	1.98	1.68	1.70		
*AT2G46730*	Pseudogene of galacturonosyl-transferase-like protein		1.31	1.33	1.34	1.16		
*AT3G42658*	Sadhu non-coding retrotransposon 3-2		5.49	5.22	5.07	5.33		
*AT3G51180*	Zinc finger C-x8-C-x5-C-x3-H type family protein ATC3H45		1.36	1.61	1.14	1.45		
*AT1G45130*	Beta-galactosidase 5 (Bgal5)	Cell wall-related biological processes	− 1.18	− 1.01	− 1.14	− 1.21	− 1.16	− 1.33
*AT1G48490*	Incomplete root hair elongation 3 (Ire3)	Controlling root skewing and maintaining the microtubule network	− 2.27	− 2.32	− 2.23	− 2.19		
*AT1G52150*	Athb-15	Vascular development	− 1.36	− 1.23	− 1.38	− 1.54		− 1.88
*AT2G34660*	Multidrug resistance-associated protein 2	Arsenite-phytochelatin transporter	− 1.42	− 1.53	− 1.54	− 1.52		1.53
*AT3G23085*	Transposable element gene		− 1.06	− 1.06	− 1.18	− 1.27		
*AT3G29642*	Transposable element gene		− 1.60	− 1.59	− 1.50	− 1.51		
*AT3G29644*	Short open reading frame 29 (Sorf29)	Natural antisense transcript overlaps with AT3G29642	− 2.82	− 2.59	− 2.87	− 2.41		
*AT3G30700*	Transposable element gene		− 1.44	− 1.21	− 1.51	− 1.42		
*AT3G43340*	RSUA2	Pseudouridine (Ψ) to uridine (U) conversion	− 2.37	− 1.95	− 2.06	− 2.14		
*AT3G43350*	Transposable element gene		− 2.97	− 2.73	− 2.84	− 2.90		
*AT3G44690*	Hypothetical protein		− 1.95	− 2.09	− 2.02	− 1.98		
*AT5G37400*	Hypothetical protein		− 1.28	− 1.20	− 1.07	− 1.14		
Important plant development related genes								
* AT1G75450*	Cytokinin Oxidase 5 (CKX5)	Degrade cytokinin			− 1.03			
* AT2G21050*	Like AUXIN RESISTANT 2 (LAX2)	Auxin influx carrier			− 1.03	− 1.06	− 1.03	-1.83
* AT4G34610*	BEL1-like homeodomain 6 (BLH6)	Negative regulator of xylem vessel cells			− 1.09	− 1.01		− 2.2
* AT3G25717*	ROTUNDIFOLIA like 16 (RTFL16)				− 1.09		3.34	− 3.4
* AT3G50870*	MONOPOLE (MNP)	Regulates shoot apical meristem and flower development			− 1.02			
* AT1G74890*	Response regulator 15 (ARR15)	Negative regulator in cytokinin-mediated signal transduction			− 1.00			
* AT3G54340*	APETALA 3 (AP3)	Specifies petal and stamen identities		1.00				
* AT5G15800*	SEPALLATA1 (SEP1)	Involved in flower and ovule development		1.52		1.00		
* AT3G02310*	SEPALLATA2 (SEP2)	Involved in flower and ovule development		1.15			− 1.10	
* AT4G18960*	AGAMOUS (AG)	Specifies floral meristem and carpel and stamen identity		1.22				
* AT5G51870*	AGAMOUS-like 71 (AGL71)	Involved in floral transition			1.07	1.03		− 1.03
* AT1G20700*	WUSCHEL related homeobox 14 (WOX14)	Regulate plant vascular proliferation				1.34		
* AT2G27220*	BEL1-like homeodomain 5 (BLH5)				1.27		5.36	1.85

Among these genes, *Beta-Galactosidase 5* was down-regulated at all time-points and was also found to be down-regulated in miR165a cell-types, compared with other cell-types (Table 2). *Beta-Galactosidase* genes are mainly involved in cell wall-related biological processes. The Arabidopsis genome encodes 17 members of this family with distinct expression patterns, suggesting non-overlapping function^19^. Further analysis of all the DEGs through heatmap visualization revealed that most DEGs have similar expression patterns across all four time points, i.e. up-regulated genes are consistently up-regulated at all the time-points and down-regulated genes are consistently down-regulated at all the time-points, although not all genes show a significant change over all time points (Fig. 2B). The smallest number of DEGs and fold-change values were found to be associated with the earliest time-point (4 h) after miR165a induction, while both the number of DEGs and fold change subsequently increased (Table 1, Fig. 2A,B).

Earlier, Zhou et al.^9^, identified *miR165a*-regulated genes in one-week old seedlings through microarray analysis after continuous over-expression of *miR165a*. Comparison of these results with this study identified 32 genes (23 up-regulated and 9 down-regulated) common between both studies (Supp Table 2). The DEGs identified in this study were also compared with our earlier RNA-Seq study performed 8 and 16 h after *miR165a* over-expression in SAM^12^, and almost one-third (up-regulated genes) to half (down-regulated genes) of the genes identified in this study were common (Supp Table 2).

To investigate the function of identified DEGs, Gene Ontology (GO) enrichment analysis was performed and GO terms associated with various biological processes were identified (Fig. 2C). Multiple GO terms related to RNA processing and cell cycle were enriched in gene sets upregulated and down-regulated, respectively, at 4 h. More interestingly, phospholipid metabolism related GO terms were enriched in down-regulated gene sets at multiple time points (8 h, 12 h, 16 h). Concerning plant development, GO terms and genes related to organ morphogenesis, such as *Cytokinin Oxidase 5 (CKX5), Like AUXIN RESISTANT 2 (LAX2), BEL1-like homeodomain 6 (BLH6), ROTUNDIFOLIA like 16 (RTFL16), MONOPOLE (MNP)* and *Response Regulator 15 (ARR15)*, were enriched in the 12 h/16 h down-regulated gene-sets. GO terms and genes related to reproduction and flower development, such as *APETALA 3 (AP3), SEPALLATA1 (SEP1), SEPALLATA2 (SEP2)* and *AGAMOUS (AG)* were enriched in the gene-set up-regulated at 8 h (Fig. 2C and Table 2). Notably, terpenoid related GO-terms were enriched in the 12 h up-regulated gene set, and plant defence response/biotic stimulus related GO-terms were enriched in genes up-regulated at 16 h. Although the role of *miR165a* is well known in plant development, our GO analysis suggests an additional role for *miR165a* in other biological processes including phospholipid metabolism, terpenoid biosynthesis and plant defence. Although a direct role of *miR165a* is not reported in pathogen defence, earlier studies have demonstrated a role for the *miR165a* target gene *REV* together with *WRKY53* acting in pathogen defence and plant immunity^20^.

### Identification of *miR165a* domain expressed genes in SAM

**Figure 3:**
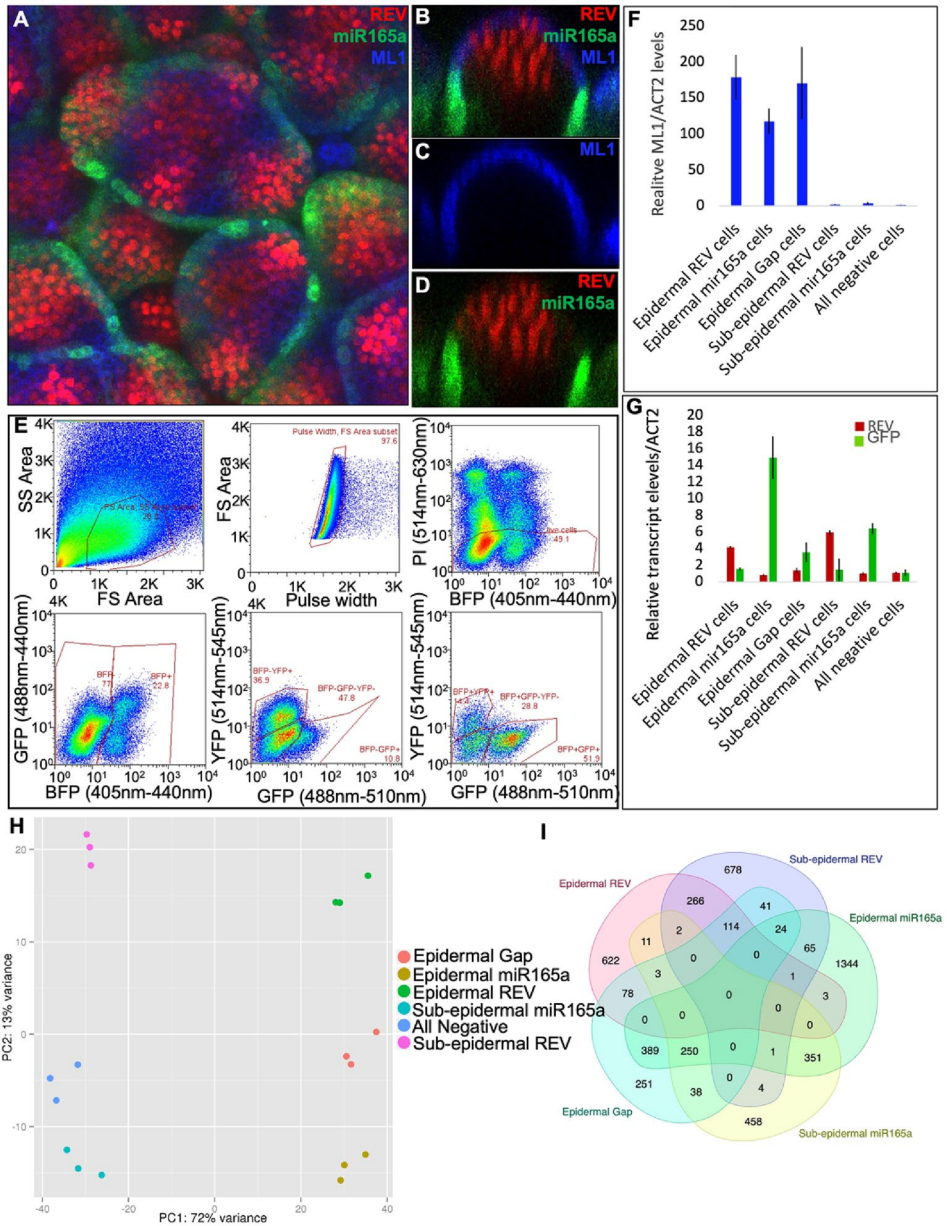
Experimental set-up for identification of genes differentially expressed in miR165a SAM domain. (**A**–**D**) Expression of *pREV*::*REV-2YPET* (red), *pmiR165a*:*GFP-ER* (green) and *pAtML1*::*mTag-BFP-ER* (blue) markers in an *ap1cal* mutant SAM. (**A**) Confocal Z-Stack top view of SAM showing expression of all three markers. (**B**) Longitudinal section view of the SAM showing all the three markers. (**C**) Longitudinal section view of the SAM showing only *AtML1* marker. (**D**) Longitudinal section view of the SAM showing *REV* and *miR165a* markers. (**E**) FACS sorting of Propidium Iodide (PI) stained protoplasts from the triple marker line. Total protoplasts were first resolved by Forward Scatter (FS) and Side Scatter (SS), and then all singlets were determined by FS Area vs pulse width analysis. Singlet protoplasts were analysed for presence of PI and BFP signals. All the PI negative protoplasts were first separated into BFP positive and BFP negative. Then, BFP positive and BFP negative protoplasts were separated into YFP positive, GFP positive and GFP&YFP negative. (**F**) Q-PCR analysis of *ML1* transcript levels in FACS sorted protoplast populations. (**G**) Q-PCR analysis of *REV* and *GFP* transcript levels in FACS sorted protoplast populations. Q-PCR experiments are normalized to reference gene *ACT2*. (**H**) Principle Component Analysis of RNA-Seq data experiment performed on the six FACS sorted protoplast populations. (**I**) Overlap analysis of specific cell-type enriched genes. For this purpose, all the genes showing significant up-regulation in an individual cell-types against the All Negative cell-type were used for overlap analysis.

In the SAM, *miR165/166* is expressed in the peripheral region, thereby restricting the expression of its targets, the *class III HD-ZIP* genes, to a more central domain^4^. To examine the spatial distribution of *miR165a* regulated genes in the SAM, we established a FACS based transcriptomics approach in which a comparison is made between genes expressed in *miR165a* expressing cells versus *miR165a* non-expressing cells in the epidermal and sub-epidermal layers of the SAM. For this purpose, three fluorescence reporter cassettes, namely *pAtML1::mTag-BFP-ER, pREV::REV-2YPET* and *pmiR165a::GFP-ER*, were introduced into the *ap1cal* mutant background for bulk collection of meristem tissues. Expression of these transgenes in SAM was confirmed by confocal imaging (Fig. 3A–D). The *pAtML1::mTag-BFP-ER* reporter marked all the epidermal cells, whereas *pREV::REV-2YPET* and *pmiR165a::GFP-ER* marked the central and peripheral part of the meristem, respectively. Meristems from the triple marker line were used for protoplast isolation and six different types of cells were collected through FACS (Fig. 3E).

**Figure 4:**
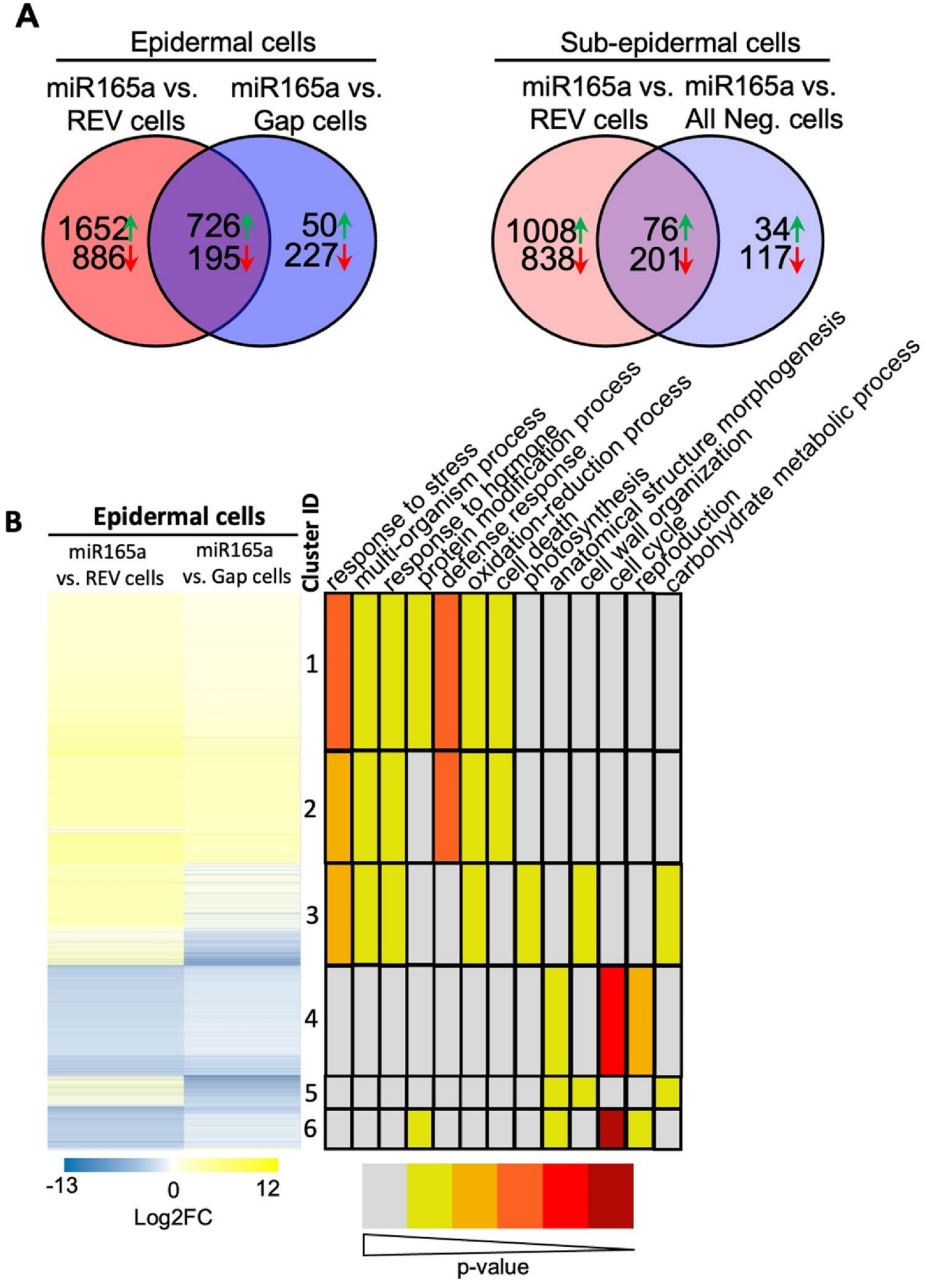
Identification of miR165a cell-type expressed genes and their GO enrichment analysis. (**A**) Overlap analysis of differentially expressed genes in miR165a marked cell-types compared with other cell-types. Green arrows represent up-regulated genes, and red arrows represent down-regulated genes. (**B**) Heatmap representation of log_2_FC in miR165a cell-type versus other cell-types in epidermis. Alongside the log_2_FC heatmap, enriched GO terms in individual cluster has been shown. The color coding for each GO term in respective cluster indicate p-value.

The six cell-types, three epidermal and three sub-epidermal, were as follows: (1) Epidermal REV cells collected on the basis of both REV and ML1 marker expression, (2) Epidermal miR165a cells collected on the basis of both miR165a and ML1 marker expression, (3) Epidermal cells collected on the basis of presence of the ML1 marker and absence of REV and miR165a markers (for easy reference, hereafter termed ‘epidermal gap’ cells, as they are present in the gap region between the REV and miR165a expression domains in the epidermis) (Fig. 3B), (4) Sub-epidermal REV cells collected on the basis of expression of the REV marker only, (5) Sub-epidermal miR165a cells, collected on the basis of only the miR165a marker expression, (6) Sub-epidermal cells collected on the basis of absence of any marker (for ease of reference, hereafter, termed ‘all negative’ cells). The purity of sorted cells was confirmed by extracting RNA from the FACS collected cell populations and measuring the expression of several cell-type specific genes using Q-PCR (Fig. 3F,G). RNA-Seq experiments were then performed to identify genes expressed in the miR165a domain as well as genes excluded from the miR165a domain. Principal Component Analysis (PCA) of RNA-Seq data revealed close clustering of the biological replicates from the same sample as well as clear separation of individual cell-types (Fig. 3H). Interestingly, the PCA revealed that the highest variation was present between epidermal versus sub-epidermal cell-types. Furthermore, it revealed that within the epidermal/sub-epidermal cell-type groups, based on transcriptome differences, REV and miR165a cells are furthest apart and that the Gap/all negative cells are in between the REV and miR165a cells in the group. Thus, the transcriptome data also reflects the spatial distance between different cell-types.

### Functional annotation of genes differentially expressed in the miR165a cell-type

To identify genes differentially expressed between the six cell-types, pair-wise DEG analyses were performed between different cell-types using a criteria of log2 fold change (FC) greater than 1 and an adjusted p-value of less than 0.05 (Supp Table 3). From these DEGs, cell-type enriched genes were identified (Supp Table 4). Gene expression was considered enriched in a particular epidermal cell-type if it was up-regulated in that cell-type against any of the remaining two epidermal cell-types. The same approach was also applied to the sub-epidermal cell-types. Overlap analysis was then performed to identify genes enriched in expression in more than one cell-type (Fig. 3I). The greatest overlap in enriched genes for the epidermal REV and epidermal miR165a cell-types was observed with their respective sub-epidermal cell-type enriched genes. Epidermal gap cell-type enriched genes showed higher overlap with epidermal miR165a cell-type enriched genes compared to epidermal REV cell-type enriched genes (Fig. 3I). For further analysis of RNA-Seq data, we mainly focused on the DEGs in miR165a cell-types. Furthermore, since there was a large difference generally between epidermal and sub-epidermal cell-types, all the subsequent analyses were done separately for epidermal and sub-epidermal cell-types. Of all the genes showing differential expression in epidermal/sub-epidermal miR165a cells, a good proportion showed differential expression compared to both REV and gap/all negative cells, though the largest proportion showed differential expression against REV cells (Fig. 4A). Heatmap visualization of genes differentially expressed in the epidermal miR165a domain reveals that except for genes present in cluster 3 and cluster 5 genes, most other genes show similar trend in expression against REV and gap cells. In other words, genes up-regulated in miR165a cells are up-regulated against both REV and gap cells, and genes down-regulated in miR165a cells are down-regulated against both REV and gap cells (Fig. 4B). However in sub-epidermal tissues, an opposite trend was observed where genes up-regulated in miR165a cells compared to REV cells were found to be down-regulated in the miR165a cells compared to all negative cells, and vice-versa (Supp Fig. 1). This suggests an expression gradient of these genes from all negative cells to miR165a cells to REV cells.

To explore the function of the miR165a domain DEGs, GO enrichment analysis was performed on the identified clusters of genes (Fig. 4B, Supp Fig. 1). In the epidermis, gene clusters up-regulated in miR165a cells compared to both REV and gap cells show enrichment for various GO terms including response to stress, defence response and cell death (Cluster 1&2, Fig. 4B). Genes present in cluster 3 and cluster 5 exhibited the following expression pattern: Gap cells > miR165a cells > REV cells, and were enriched for GO terms such as carbohydrate metabolic process and cell-wall organization (Cluster 3&5, Fig. 4B). These two clusters represent around 1000 genes, including some important cell-wall related genes such as *XYLOGLUCAN ENDOTRANSGLUCOSYLASE/HYDROLASEs, PECTIN METHYLESTERASE, PECTIN METHYLESTERASE INHIBITORs,* various *EXPANSIN and EXTENSINs*, and development related genes such as *LIKE AUX1 3, PINOID2, AINTEGUMENTA-LIKE 5, GH3.4 , APETALA 3, SEPALLATA 3, PETAL LOSS* and *PERIANTHIA.* GO terms related to reproduction, cell-cycle and plant development were found to be enriched in gene clusters showing down-regulation in miR165a cells against both REV and gap/all negative cells as well as in sub-epidermal cluster 7, which represents genes down-regulated in miR165a cells against the REV cells, but up-regulated in miR165a cells against the all negative cells. (Cluster 4&6, Fig. 4B; Cluster 6, Supp Fig. 1).

In terms of genes that show differential expression specific to the miR165a SAM domain compared to all other tissues, we found 33 genes that were up-regulated (enriched) and 15 genes down-regulated (excluded) in both epidermal and sub-epidermal miR165a cell-types against all other non-miR165a cell-types (Table 3).

**Table 3:** Genes up-regulated or down-regulated in both epidermal and subepidermal miR165a cell-types against all other non-miR165a cell-types.

Gene ID	Given name	Functions
Up-regulated in both epidermal and sub-epidermal miR165a cell-types against all other cell-types		
* AT1G63360*		Disease resistance protein (CC-NBS-LRR class) family
* AT5G20050*		Protein kinase superfamily protein
* AT1G05135*		Pseudogene
* AT1G16930*		F-box/RNI-like/FBD-like domains-containing protein
* AT2G05380*		Glycine-rich protein 3 short isoform (GRP3S)
* AT1G67750*		Pectate lyase family protein
* AT2G16895*		Pseudogene of UDP-Glycosyltransferase superfamily protein
* AT4G14746*		Neurogenic locus notch-like protein
* AT4G18220*		Drug/metabolite transporter superfamily protein
* AT2G18210*		Hypothetical protein
* AT4G18250*		Receptor Serine/Threonine kinase-like protein
* AT5G57123*		Hypothetical protein
* AT5G46490*		Disease resistance protein (TIR-NBS-LRR class) family
* AT1G30730*	*ATBBE11*	FAD-binding Berberine family protein
* AT5G24210*	*PRLIP1*	Alpha/beta-Hydrolases superfamily protein
* AT3G55240*	*RPGE3*	Overexpression leads to PEL (Pseudo-Etiolation in Light) phenotype
* AT1G23090*	*SULFATE TRANSPORTER 91 (AST91)*	Encodes for sulfate transporter
* AT5G15160*	*BANQUO 2 (BNQ2)*	Required for appropriate regulation of flowering time
* AT1G29290*	*C-TERMINALLY ENCODED PEPTIDE 14 (CEP14)*	Binds to vascular tissue independently of CEPR1 or CRA2
* AT1G19670*	*CHLOROPHYLLASE 1 (CLH1)*	Involved in chlorophyll degradation
* AT3G26200*	*CYTOCHROME P450, FAMILY 71, SUBFAMILY B, POLYPEPTIDE 22 (CYP71B22)*	Putative cytochrome P450
* AT3G25180*	*CYTOCHROME P450, FAMILY 82, SUBFAMILY G, POLYPEPTIDE 1 (CYP82G1)*	Catalyzes the production of two volatile homoterpenes, TMTT and DMNT
* AT3G50480*	*HOMOLOG OF RPW8 4 (HR4)*	
* AT5G39020*	*MEDOS 3 (MDS3)*	Involved in growth adaptation upon exposure to metal ions
* AT5G39030*	*MEDOS 4 (MDS4)*	Involved in growth adaptation upon exposure to metal ions
* AT5G26230*	*MEMBRANE-ASSOCIATED KINASE REGULATOR 1 (MAKR1)*	
* AT3G45290*		MILDEW RESISTANCE LOCUS O 3 (MLO3)
* AT4G18197*	*PURINE PERMEASE 7 (PUP7)*	Involved in the transport of cytokinins
* AT5G52250*	*REPRESSOR OF UV-B PHOTOMORPHOGENESIS 1 (RUP1)*	Functions as a repressor of UV-B signaling
* AT4G23870*	*TRANSITION ZONE (TZ1)*	Mutants have increased resistance to Al
* AT2G29330*	*TROPINONE REDUCTASE (TRI)*	
* AT1G08465*	*YABBY2*	Involved in the abaxial cell fate specification in lateral organs
* AT1G25250*	*IDD16*	Regulates auxin biosynthesis and transport aerial organ morphogenesis
Down-regulated in both epidermal and sub-epidermal miR165a cell-types against all other cell-types		
* AT1G80100*		Acts as an inhibitor of cytokinin signaling
* AT1G49475*		AP2/B3-like transcriptional factor family protein
* AT1G68630*		PLAC8 family protein
* AT4G24050*		NAD(P)-binding Rossmann-fold superfamily protein
* AT5G23100*		MIZU-KUSSEI-like protein
* AT3G02000*	*ROXY1*	It is required for proper petal initiation and organogenesis
* AT5G43810*	*ZWILLE (ZLL)/ARGONAUTE 10 (AGO10)*	specifically sequesters miR166/165
* AT1G13710*	*KLUH (KLU)*	Contributes to growth-stimulating signal
* AT5G43870*	*FORKED-LIKE1 (FL1)*	Coordinate leaf size with vein density
* AT1G10540*		NUCLEOBASE-ASCORBATE TRANSPORTER 8 (NAT8)
* AT3G50410*	*OBF BINDING PROTEIN 1 (OBP1)*	Play an important roles in plant growth and development
* AT1G73590*	*PIN-FORMED 1 (PIN1)*	Auxin efflux carrier
* AT4G31620*	REPRODUCTIVE MERISTEM 36 (REM36)	
* AT4G25810*	*XYLOGLUCAN ENDOTRANSGLYCOSYLASE 6 (XTR6)*	
* AT5G11320*	*YUCCA4 (YUC4)*	Part of a pathway linking auxin biosynthesis

Log2FC > 1 and adjusted p-value < 0.05 were used as a criteria for DEG consideration.

Among the miR165a domain enriched genes, plant development related genes *YABBY2, IDD16, PURINE PERMEASE 7 (PUP7), C-TERMINALLY ENCODED PEPTIDE 14 (CEP14)* and *BANQUO 2 (BNQ2)* were present*.* We also found genes known to be involved in metal homeostasis *(SULFATE TRANSPORTER 91 (AST91), MEDOS 3, MEDOS 4, TRANSITION ZONE 1 (TZ1))* and light-signalling *(REPRESSOR OF UV-B PHOTOMORPHOGENESIS 1 (RUP1), RPGE3, CHLOROPHYLLASE 1 (CLH1)* were also enriched in miR165a cells. However, most of the miR165a domain-excluded genes, such as *YUCCA4, PIN1, OBF BINDING PROTEIN 1 (OBP1), KLUH (KLU), FORKED-LIKE1 (FL1), ZWILLE (ZLL)/ARGONAUTE 10 (AGO10),* and *ROXY1* (Table 3) are related to only plant development.

### Integration of miR165a-regulated genes and genes differentially expressed in miR165a SAM domain

**Figure 5:**
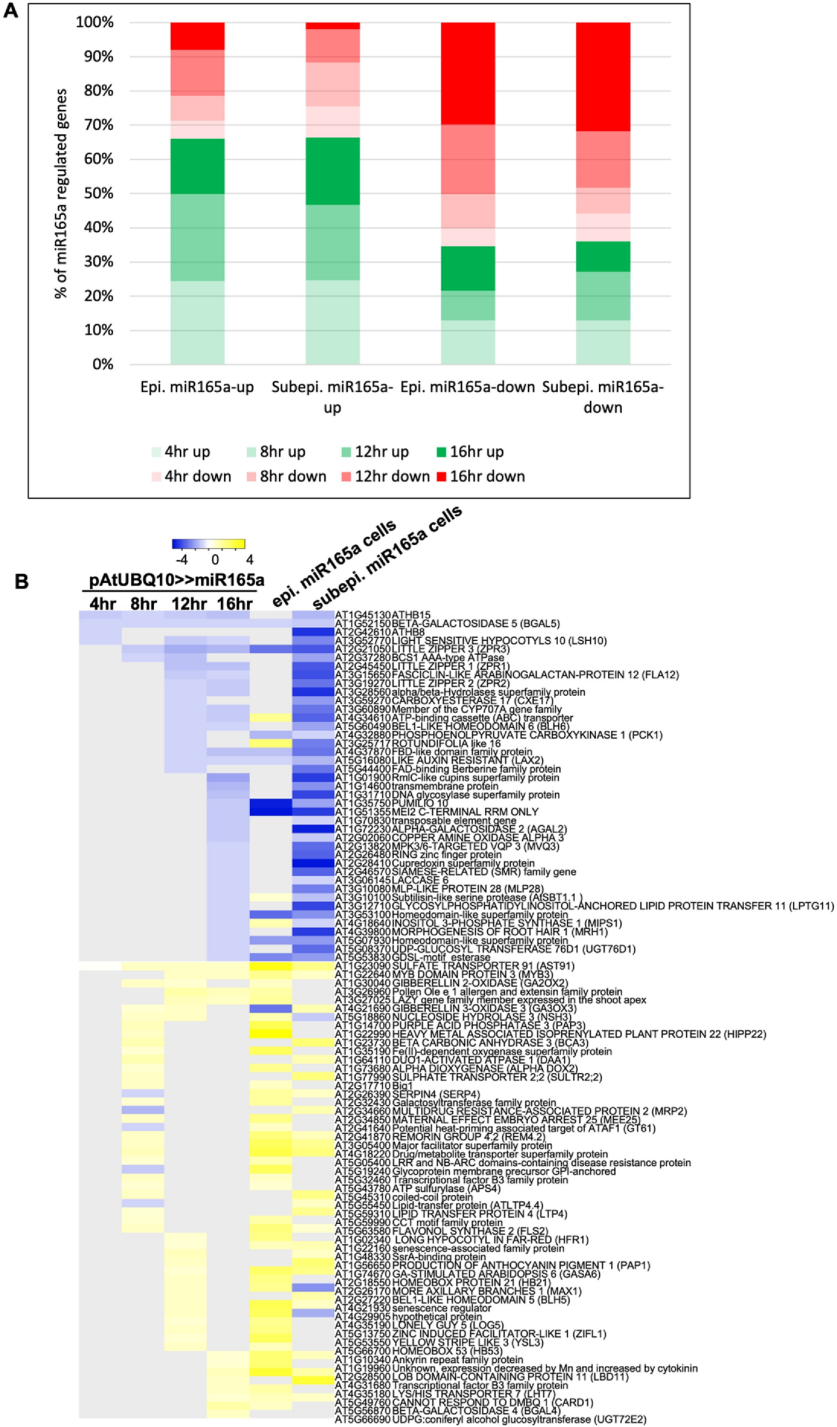
Integrated analysis of both RNA-Seq experiment. (**A**) Each stacked bar represents proportion of overlapping genes between the set of genes up/down-regulated in epidermal/subepidermal miR165a cells against respective epidermal/subepidermal non-miR165a cells, and genes regulated by miR165a at different time points to total number of miR165a up/down regulated genes at particular time point. (**B**) Heatmap visualization of Log2FC of genes which are significantly affected and show same trend in expression in both sets of RNA-Seq experiments (over-expression of miR165a and cell-type profiling). The Log2FC of miR165a cell-types against REV cell-types has been plotted here. The grey colour indicate that gene is not significantly changed in particular time-point or cell-type.

Having identified genes regulated by *miR165a* in SAM as well as genes differentially expressed in the miR165a SAM domain compared to other cell types, we next examined the overlap between these two datasets. For this purpose, we examined the extent of overlap of genes up or downregulated by *miR165a* at different time points, with genes identified to be expressed differentially in the epidermal/subepidermal miR165a SAM domains (Fig. 5A). We found that within the set of genes expressed at relatively high levels in the miR165a domain, genes up-regulated by *miR165a* account for a greater proportion of overlap compared to genes down-regulated by *miR165a* (Fig. 5A). Similarly, within the set of genes down-regulated in the miR165a domain, a larger overlap exists with the set of genes down-regulated by *miR165a* than genes up-regulated by *miR165a*. In an alternative approach, the expression of *miR165a* regulated genes at different time points was examined with respect to epidermal/subepidermal miR165a cells, and it was observed that a large proportion of genes up-regulated at different time-points show higher expression in miR165a cells while a large proportion of down-regulated genes show lower expression in miR165a cells (Fig. 5B). Thus both types of overlap analysis show a similar trend. Among the genes which are up-regulated by miR165a as well as have enriched expression in miR165a cells, *MIR165a, SULFATE TRANSPORTER 91 (AT1G23090), GIBBERELLIN 3-OXIDASE 1 (AT1G15550), MYB DOMAIN PROTEIN 3 (AT1G22640)* were consistently up-regulated across multiple time-points after *miR165a* induction (Fig. 5B). Additionally, iron and zinc transporter genes such as *YELLOW STRIPE LIKE 3 (AT5G53550)* and *ZINC INDUCED FACILITATOR-LIKE 1 (AT5G13750)* were also present in this category. In the second category of genes which are down-regulated by miR165a and show comparatively lower expression in miR165a cells, *HD-ZIPIII* genes *(ATHB8 and ATHB15)*, and HD-ZIPIII downstream target genes (three *LITTLE ZIPPERs*) are present (Fig. 5B). Additionally, some other important development related genes, such as *ARABIDOPSIS RESPONSE REGULATOR 15 (ARR15), LIKE AUXIN RESISTANT 2 (LAX2), MONOPOLE (MNP)* were also identified in this category. Thus, the integration of results from both RNA-Seq experiments helped to identify highly probable targets of *miR165a* and *HD-ZIPIII* genes involved in plant developmental as well as in other biological processes.

## Discussion

Both *miR165* and *miR166* family members repress *HD-ZIPIII* gene expression in abaxial leaf tissues as well as in the periphery of the SAM^1–5^. HD-ZIPIII TFs are direct targets of these miRNAs; thus, the biological role of these miRNAs is directly correlated with the function of *HD-ZIPIII* genes. *HD-ZIPIII* genes play an important role in leaf polarity, new organ initiation, vasculature development, SAM maintenance, and shade avoidance^4,21^. Additionally, the role for *miR165/166* has also been reported in the regulation of ABA levels and abiotic stress response, particularly in relation to drought and cold stress, through *HD-ZIPIII* genes. Thus, given the diverse role of the *miR165/166* and *HD-ZIPIII* genes, this work aimed to understand the global gene regulatory network underlying *miR165a* and *HD-ZIPIII* genes in Arabidopsis using RNA-Seq approaches.

In our earlier study^12^, although we had identified *miR165a* regulated DEGs, however genes differentially expressed in miR165a domain were not identified. Furthermore, in this previous study, the number of *miR165a* regulated DEGs was quite low, e.g. only 150 genes were identified as DEGs at 8 h. So, here in this study we identified genes differentially expressed in miR165a domain in SAM through FACS-based RNA-Seq approach, as well as performed new experiments to identify *miR165a* regulated DEGs. For identifying *miR165a* regulated DEGs, we used the *GR-LhG4* system and *miR165a* was over-expressed in temporal manner (4, 8, 12 and 16 h) to capture the temporally dynamic transcriptome of SAM. In the GR-LhG4 system, LhG4 is a transcriptional activator, that binds to operator DNA sequences^14^. To control the effect of LhG4 on transcription in our study, we generated the *pAtUBQ10::LhG4* line in the same genetic background, *ap1cal*, and used this line along with *pAtUBQ10*>>*miR165a* in *ap1cal* for RNA-Seq analysis. Comparing the transcriptome in the *pAtUBQ10::LhG4* line against the background line *ap1cal* confirmed that indeed the LhG4 binds non-specifically in the Arabidopsis genome and activates the expression of handful number of genes (Table 1). Thus, for the identification of true *miR165a* regulated genes, the transcriptome of *pAtUBQ10*>>*miR165a* in *ap1cal* was compared against the *pAtUBQ10::LhG4* in *ap1cal*.

The number of *miR165a* regulated DEGs identified in this study is even smaller than in our previous study^12^, where less than 450 DEGs were identified, suggesting that indeed through this approach only a low number of DEGs could be identified. Furthermore, the comparison of *miR165a* regulated genes identified in this study with REV regulated genes from our previous study^12^ showed only a handful number of overlapping genes (Fig. S2). One reason behind this could be the fact that for identification of REV regulated genes, miR165/166 resistant *REV* (*REVr)* was ectopically expressed in the epidermis *(AtML1*>>*REVr)* followed by FACS sorting of epidermal cells and RNA-Seq. Whereas for the identification of *miR165a* regulated genes, *miR165a* was expressed under the *AtUBQ10* promoter followed by RNA extraction from the whole SAM tissue. These results suggest that FACS sorting of the cell-types increases the sensitivity of the RNA-Seq experiments. In the future, ectopic expression of *miR165a* in the epidermis *(pAtML1*>>*miR165a)* followed by FACS sorting of epidermal cells and RNA-Seq would be a better experiment for comparison with REV regulated genes identified from *pAtML1*>>*REVr* lines.

Comparison of *miR165a* regulated genes identified in this study and our previous study^12^, revealed only around 50% overlap of DEGs. Given the fact that both the studies were performed using the same transgenic lines, same laboratory conditions, and the same analysis pipeline, we conclude that in our hands RNA-Seq studies have only around 50% reproducibility. Previous systematic analysis of multiple RNA-Seq studies also revealed low reproducibility of RNA-Seq data^22,23^. Although, there was little overlap between significant DEGs at different time-points, however, most of the DEGs showed a similar expression pattern (either up or down-regulation) across all four time points (Fig. 1A,B). This suggests that at each time-point only specific genes show significant changes in their expression, but at other time points, those genes show the same expression pattern, but their fold change expression is non-significant. Most of the GO terms enriched in 4 h up-regulated genes were related to RNA modification and processing. This is not unexpected, as there is high expression of *miR165a* at the early time-point and the plant may activate the machinery of RNA processing. GO terms enriched in 4 h down-regulated genes were mostly related to the cell-cycle. As the collected tissue was SAM, which was undergoing rapid cell-division, and it perceived miR165a induction as a stress and, thus stopped its normal activity (cell-division), and up-regulated stress response machinery. The ZPR genes, which are direct targets of the HD-ZIPIII target genes, are not affected at the early time-point, but are down-regulated only at later time-points (12 and 16 h). This suggests that DEGs identified at later time-points are more likely to target genes of *HD-ZIPIIIs*. *HD-ZIPIIIs* have been shown to repress organ formation in centre of the meristem^4^, and as expectedly, our results show GO terms related with floral organ development are enriched in 8 h up-regulated genes.

To identify the genes differentially expressed in the miR165a cells, we performed RNA-Seq analysis on cells sorted on the basis of fluorescence signals of miR165a and REV reporters. Addition of third reporter, *AtML1*, further helped to separate miR165a and REV cells into epidermal and non-epidermal cells. Q-PCR analysis of marker genes on the sorted cells has confirmed the efficient sorting of all six-type of protoplasts. Similarly, PCA plot analysis revealed separate clustering of each cell-type, confirming the proper sorting of different cell-types. Most of the further analysis of cell-type profiling data was mainly focused on miR165a cells. This analysis identified genes with enriched/depleted expression in miR165a cells compared with other cell-types. These genes included gene-sets which are exclusively enriched only in miR165a cells but not in any other cell-types, genes enriched in miR165a cells against all other cell-types, genes depleted in miR165a cells but expressed in all other cell-types and, so on.

The identification of miR165a and REV (a HD-ZIPIII) cell-types specific genes and their integration with *miR165a* regulated genes (which also represent HD-ZIPIII regulated genes) in the same tissue (SAM) provides the opportunity to find-out the most relevant genes in this study. Our results show that among the genes up-regulated by *miR165a*, the majority have higher expression in miR165a cells, and among the genes down-regulated by *miR165a*, majority have higher expression in non-miR165a cells. However, it is worth mentioning here that such genes are not necessarily target genes of the HD-ZIP III; they could be targets of other adaxial genes. Thus, identification of relevant *miR165a* regulated genes through integration of both the RNA-Seq experiments reveals novel role of *miR165a* in different biological processes. Further phenotypic analysis of knock-out/over-expression lines of *miR165a/HD-ZIPIII* in the identified biological process would help confirm the observed role of *miR165a/HD-ZIPIII* genes.

## Materials and methods

### Plant materials and growth conditions

The *ap1cal* seeds were used in our previous study^12^, and all other plants were generated in this study. All the plants were gown at 22 °C under continuous light conditions. For *miR165a* over-expression purpose, DNA construct containing expression cassettes *pAtUBQ10*::*GR-LhG4;6OP*::*miR165a* were transformed into the *ap1cal* mutant background. As a control, DNA construct *pAtUBQ10*::*GR-LhG4* was transformed into the *ap1cal* mutant background. To generate reporter lines for FACS experiments, DNA construct *pHGW-pREV*::*REV-2YPET;pmiR165a*::*GFPer* was transformed into the transgenic line containing DNA construct *pMOA33-pAtML1*::*mTag-BFP-ER* in *ap1cal* mutant background. All the experiments were done in T2 generations in all the transgenic lines.

### Recombinant DNA constructs

For preparing the *pAtUBQ10*::*GR-LhG4* construct, a 2 kb region of the 5’ regulatory sequence from the *UBQ10* gene *(AT4G05230)* was cloned upstream to the *GR-LhG4* CDS through *BamH1* restriction digestion and ligation in *pBJ36* vector. The resulting expression cassette was then transferred to a sulfadiazine resistant T-DNA vector *pSULT* through *NotI* mediated restriction digestion and ligation. To construct the *pAtUBQ10*::*GR-LhG4;6OP*::*miR165a* DNA construct, first *6xOp::miR165a* part was constructed by putting the corresponding operator sequence (6x) of the *LhG4* upstream to the primary transcript regions of *miR165a* in *pBJ36* vector. Then the expression cassette of *6xOp::miR165a* was transferred to the T-DNA vector *pSULT* containing *pAtUBQ10*::*GR-LhG4* to achieve *pAtUBQ10*::*GR-LhG4;6OP*::*miR165a*. To create the *pMOA33-pAtML1::mTag-BFP-ER* construct, a fragment of ER-localized *mTag-BFP*^24^ was synthesized (Genscript) and cloned into the *pBJ36* vector downstream of 3.4 kb of the L1 layer-specific *AtML1* gene *(AT4g21750)* promoter. The entire expression cassette was then transferred into the T-DNA vector *pMOA33*^25^ through *NotI* restriction digestion and ligation. *pmiR165a*::*GFPer* construct is described in^26^, and *pREV*::*REV-2YPET* construct is described in^12^. Both the *pmiR165a*::*GFPer* and *pREV*::*REV-2YPET* construct were transferred to same T-DNA in *pHGW* vector using Gateway multiplexing approach.

### Dexamethasone treatment

To induce gene perturbations in the SAM, a solution of 10 μM DEX (dexamethasone) was applied to the SAM once. The solution also contained 0.015% Silwet L-77, a surfactant that helps the DEX solution to adhere to the IM. Tissues were collected after the desired time point for RNA-Seq experiments. For observing the phenotype, the DEX solution was applied every second day for a total of 7 days. Ethanol containing 0.015% Silwet L-77 was used as a mock solution for concurrently treating untreated samples.

### Protoplast preparation

For protoplasting, Buffer A was prepared by mixing the following ingredients: 10 mM KCl, 2 mM MgCl_2_, 2 mM CaCl_2_, 0.1% BSA, 2 mM MES hydrate, and 0.6 M Mannitol. The buffer was filter sterilized and stored at 4 °C for long-term use. To prepare the protoplasts, 1.5% Cellulase RS 10 and 0.1% Pectolyase Y23 were dissolved in Buffer A. SAMs from 4 to 5 week old plants were placed in the enzyme solution in a 70 µm cell strainer in a small petri dish and SAM were chopped into small pieces and incubated in the dark at 22 °C for 60 min. The protoplasts were gathered through centrifugation at 500*g* for 7 min at 4 °C. Afterward, the supernatant was cautiously eliminated to avoid disrupting the protoplast pellet. The pellet was subsequently re-suspended in 5 ml of Buffer A and centrifuged once more. The resulting supernatant was discarded, and the pellet was re-suspended in 1 ml of Buffer A. Next, the re-suspended pellet suspension was filtered through a 40 µm cell strainer in a small petri dish and utilized for sorting using a FACS.

### FACS sorting

The EMBL flow cytometry core facility employed a specialized MoFlo XDP cell sorter manufactured by Beckman Coulter, which was equipped with three lasers. These lasers included a Coherent Sabr Argon gas laser with an emission wavelength of 514.5 nm and power output of 400 mW, a Coherent Innova gas laser emitting at 488 nm with a power output of 300mW, and a Coherent Obis 405 nm solid-state laser with a power output of 240mW. The sorting process involved capturing the forward scatter (FSC), side scatter (SSC), and enhanced green fluorescent protein (eGFP) signals from the 488 nm laser, utilizing specific band-pass filters and a beam-splitter. The yellow fluorescent protein (YFP) and propidium iodide (PI) signals were detected using the 514.5 nm laser with appropriate band-pass filters and a beam-splitter. The blue fluorescent protein (BFP) signals were obtained from the 405 nm laser using a specific band-pass filter. Due to minimal spectral overlap, no compensation was required. Gating was performed based on the ratios of SSC height to area, and the sorting was carried out using FACSFlow (BD) as the sheath fluid at a pressure of 30 psi, with a 100 µm nozzle and a frequency of 40 kHz for protoplast sorting. For RNAseq library preparation, 100,000 viable protoplasts were directly sorted into 500 µl RLT buffer (Qiagen). Finally, the sorted samples were promptly frozen in liquid nitrogen, and used for total RNA extraction.

### Total RNA-extraction

The cryopreserved protoplasts were thawed at 37 °C, and the RLT buffer volume was adjusted to three times the volume of the protoplasts. Three biological replicates were used for each type of sample. The combination was mixed by vortexing and then incubated at room temperature for 5 min. Afterward, the mixture was vigorously vortexed again to lyse the protoplasts. Subsequently, the RNeasy Mini kit (Qiagen) was employed following the manufacturer's instructions. For RNA extraction from SAM tissue, 4–5 SAMs were collected from independent three biological replicates and quickly frozen in liquid nitrogen. The tissue were stored in-8-until further use. RNeasy Mini kit (Qiagen) was used for the RNA extraction.

### Q-PCR analysis

cDNA was prepared using Super script III reverse transcriptase (Thermo Scientific) enzyme. For RNA obtained from protoplast, around 25 ng total RNA was used whereas in case of SAM RNA 2 µg of total RNA was used as starting material. StepOne Plus Real Time PCR system thermo cycler, from the applied bio systems was used for Q-PCR experiment in a 20 µl reaction using 2X Syber Green master mix (Roche), 1:5 diluted cDNA and respective primers. Two technical replicate were used for each sample. Actin 2 was used as an internal reference gene. The Q-PCR data were analysed using 2^−ΔΔCT^ method.

### NGS library preparation

For preparing RNA-seq libraries from SAM RNA, “TruSeq Stranded mRNA Library Prep Kit from Illumina” was used, and 1 µg of total RNA was used. For preparing RNA-seq libraries from RNA obtained from FACS sorted protoplasts, “NEBNext Ultra RNA Library Prep Kit for Illumina” was used using 10 ng of total RNA.

### RNA-Seq data analysis

The sequencing data's quality was evaluated using FastQC. Total 16 to 38 millions reads were obtained for different samples in both RNA-Seq experiments. PCR primers, low-quality bases (with a Phred score < 20), and bases with varying GC content were removed by cutadapt^27^. To map the genome, TopHat^28^ was employed, using an index derived from the TAIR 10 genome release, and 90% of total reads were successfully mapped to reference genome. A gene expression count table was created from the aligned dataset using HTSeq^29^, utilizing TAIR 10 gene annotations and focusing solely on the exonic regions of the gene. The analysis for differential expression was conducted using the DESeq2^30^ R package. Genes with an adjusted p-value of ≤ 0.05 and log2 fold change of ≥ 1 were considered to be differentially expressed. This set of genes was then utilized for subsequent downstream processing. GO enrichment analysis was performed using AgriGo V2 tool, using Arabidopsis genome (TAIR 10) as a reference set. GO terms related with biological processes were selected based on following criteria: p-value cut-off: 0.05, minimum number of over represented genes: 5.

### Ethical approval

All procedures were conducted in accordance with the guidelines.

### Supplementary Information

Supplementary Figures.

Supplementary Table 1.

Supplementary Table 2.

Supplementary Table 3.

Supplementary Table 4.

Supplementary Table 5.

Supplementary Table 6.

Supplementary Table 7.

Supplementary Table 8.

Supplementary Table 9.

Supplementary Table 10.

Supplementary Table 11.

## Data Availability

The raw data of this study are available at Gene expression Omnibus (GEO) with accession no. GSE221139.
