# Supplementary material for: Global gene regulatory network underlying miR165a in Arabidopsis shoot apical meristem
Source: Sci Rep. 2023 Dec 14;13:22258. doi: 10.1038/s41598-023-49093-2 (PMC10721644; doi:10.1038/s41598-023-49093-2)
Supplement: Supplementary file 1 — Supplementary Figures. [file 41598_2023_49093_MOESM1_ESM.pdf]

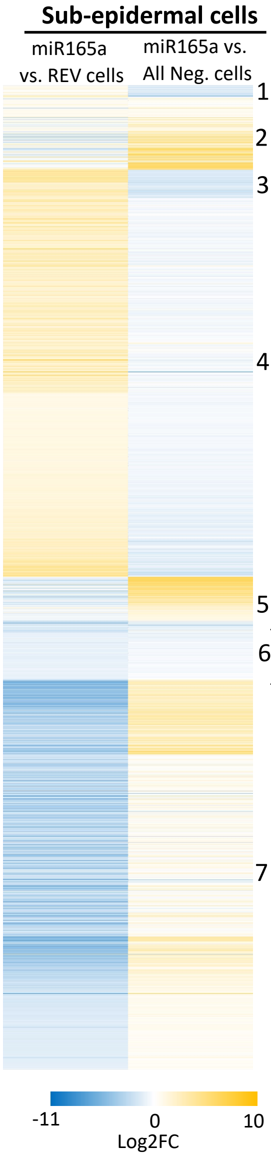

Cluster	GO term	p-value
1	oxidation-reduction process	0.042
2	ion transport	0.001
	anatomical structure development	0.002
	response to hormone	0.004
3	single-organism process	0.008
4	response to stimulus	8.4e-16
	single-organism process	1.4e-15
	oxidation-reduction process	2.4e-07
	defense response	3.6e-07
	response to hormone	5.7e-05
5	organic anion transport	0.014
	glucosinolate metabolic process	0.014
	carboxylic acid transport	0.014
6	cell cycle	1.4e-18
	tissue development	0.0011
	meristem development	0.016
7	cell cycle	3.5e-15
	tissue development	1e-10
	transcription, DNA-templated	1.2e-08
	pattern specification process	8.7e-07
	meristem development	2.5e-06

Figure S1. Heatmap representation of log_2FC in miR165a cells versus other cell-types in sub-epidermis. Alongside the log_2FC heatmap, enriched GO terms and associated p-value in individual cluster has been shown.

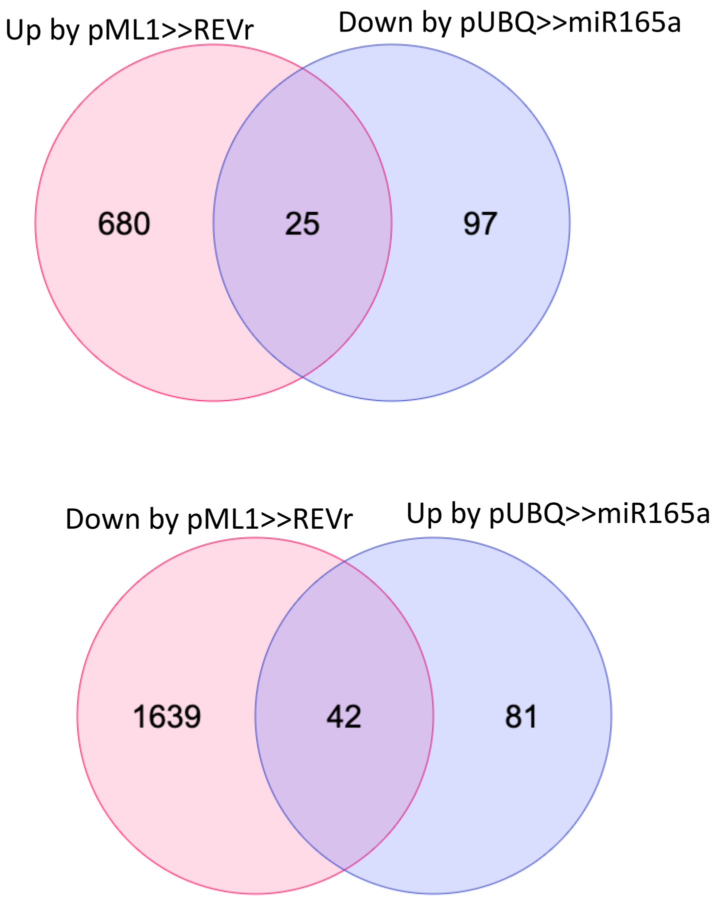

Figure S2. Overlap analysis of DEGs identified after ectopic expression of REV in epidermis (pML1>>REVr) (Ram et al., 2020), and after over-expression of miR165a (pAtUBQ10>>miR165a) in this study.
